# Editorial: Gut microbiota and gastrointestinal disorders, volume II

**DOI:** 10.3389/fmed.2025.1576152

**Published:** 2025-03-17

**Authors:** Abbas Yadegar, Aryan Salahi-Niri, Yan-Dong Wang, Javier Ochoa-Repáraz

**Affiliations:** ^1^Foodborne and Waterborne Diseases Research Center, Research Institute for Gastroenterology and Liver Diseases, Shahid Beheshti University of Medical Sciences, Tehran, Iran; ^2^State Key Laboratory of Chemical Resource Engineering, College of Life Science and Technology, Beijing University of Chemical Technology, Beijing, China; ^3^Department of Biological Sciences, Boise State University, Boise, ID, United States

**Keywords:** gut microbiota, gastrointestinal disorders, dysbiosis, gastric cancer, inflammatory bowel disease, acute pancreatitis

The human gut microbiota, an intricate ecosystem of bacteria, viruses, fungi, and archaea, plays a pivotal role in maintaining gastrointestinal (GI) homeostasis (1). Recent advances in microbiome research have revealed its profound influence on various GI diseases, such as gastric cancer, inflammatory bowel disease (IBD), irritable bowel syndrome (IBS), acute pancreatitis, and *Clostridioides difficile* infection (CDI) (2). Understanding the interplay between the gut microbiota and these conditions offers promising avenues for novel therapeutic interventions, including next-generation probiotics, fecal microbiota transplantation (FMT), and targeted microbiome-directed modulation (3, 4).

Gastric cancer remains a significant global health burden, ranking as the fourth leading cause of cancer-related mortality. Dysbiosis, characterized by altered microbial diversity and composition, is a hallmark of gastric carcinogenesis (Marashi et al.). *Helicobacter pylori* infection is a well-established risk factor, but eradication alone does not fully restore microbial equilibrium. Studies suggest that certain bacterial species, such as *Lactobacillus*, enhance the response to immunotherapy, whereas antibiotic-induced dysbiosis may reduce the efficacy of chemotherapy (Marashi et al.) (5, 6). Probiotic supplementation after gastrectomy has demonstrated the potential to mitigate inflammation and foster a healthier microbial milieu (Marashi et al.) (7).

IBDs, which include Crohn's disease and ulcerative colitis, are immune-mediated diseases driven by a complex interplay of genetic, environmental, and microbial factors (Ning et al.) (8). Dysbiosis in IBDs is marked by a reduction of beneficial Firmicutes and Bacteroidetes and an overrepresentation of pathogenic Proteobacteria and Actinobacteria (Ning et al.). The intricate crosstalk between gut microbiota and intestinal macrophages plays a crucial role in disease pathogenesis by influencing inflammation and immune responses (Ning et al.). Current therapeutic strategies, including 5-aminosalicylic acid, immunomodulators, and biologics, inadvertently alter gut microbiota composition, highlighting the need for microbiome-targeted interventions such as FMT and prebiotic supplementation (Ning et al.) (9).

IBS, a prevalent functional GI condition, is increasingly linked to microbial imbalances (Cheng et al.). Patients exhibit reduced microbial diversity and a distinct shift in gut microbiota composition, with lower *Bifidobacterium* and *Lactobacillus* levels and increased levels of Enterobacteriaceae (Cheng et al.). Notably, alterations in the gut microbiota vary based on ethnic background, as demonstrated in comparative studies between Han and Tibetan populations (Ma et al.). The distinct gut microbial profiles among ethnic groups indicate that cultural and dietary habits influence the gut microbiota and, consequently, disease susceptibility. Probiotics, prebiotics, and dietary modifications have shown efficacy in restoring microbial equilibrium and alleviating IBS symptoms (Cheng et al.).

Acute pancreatitis (AP) is an inflammatory condition with rising global incidence. The gut microbiota plays a critical role in AP pathogenesis through mechanisms involving increased intestinal permeability, bacterial translocation, and systemic inflammation (Li et al.). Dysbiosis in AP is associated with severe complications, including multiple organ dysfunction syndrome (10). Innovative therapies, such as gut microbiota-derived extracellular vesicles, show promise in mitigating AP severity by modulating immune responses (Li et al.). By targeting the underlying microbial imbalances, these therapeutic approaches may significantly improve patient outcomes in AP management (11).

CDI, a leading cause of healthcare-associated diarrhea, is predominantly driven by antibiotic-induced gut dysbiosis (Duo et al.). The loss of microbial diversity facilitates *C. difficile* colonization, leading to recurrent infections in a significant proportion of patients. FMT has emerged as the most effective therapy for recurrent CDI (rCDI), surpassing antibiotics in efficacy by restoring a balanced gut microbiome (Duo et al.) (12). Despite its success, concerns regarding standardization and safety necessitate further research into microbiota-based therapeutics (Duo et al.).

The intricate relationship between the gut microbiota and metabolic functions extends beyond the GI tract. Emerging research has explored the bidirectional connection between the gut microbiota and insulin-like growth factor 1 (IGF-1), a key regulator of metabolic and growth-related pathways (Zheng et al.). Dysbiosis has been implicated in modulating IGF-1 levels, suggesting potential metabolic consequences of gut microbial imbalances. This finding broadens the scope of microbiome-targeted therapies to include metabolic disorders alongside GI diseases.

The expanding knowledge on the role of gut microbiota in GI disorders underscores the need for precision medicine approaches. Personalized microbiome profiling could guide tailored interventions to optimize therapeutic outcomes. Probiotics, prebiotics, synbiotics, and next-generation microbiome-based therapies, including engineered bacterial consortia and postbiotics, offer exciting prospects. Moreover, advances in metagenomics and metabolomics will further elucidate host-microbe interactions, paving the way for novel therapeutic strategies (13).

In conclusion, the published studies in this Research Topic highlight the intricate role of the gut microbiota as an integral determinant of human GI health and disease. From gastric cancer to IBS and IBD, the influence of the gut microbiome is profound and multifaceted (Stange et al.). Harnessing microbiome-based interventions holds immense potential to revolutionize the management of GI conditions, shifting from symptomatic treatment to root-cause modulation (14). As research continues to unravel the complexities of the gut microbiota, the future of gastroenterology lies in leveraging this microscopic powerhouse for transformative healthcare solutions.

## References

[1] Joos R Boucher K Lavelle A Arumugam M Blaser MJ Claesson MJ. Examining the healthy human microbiome concept. Nat Rev Microbiol. (2025) 23:192–205. 10.1038/s41579-024-01107-0 39443812

[2] Yaqub MO Jain A Joseph CE Edison LK. Microbiome-driven therapeutics: from gut health to precision medicine. Gastrointest Disord. (2025) 7:7. 10.3390/gidisord701000737419381

[3] Yadegar A Bar-Yoseph H Monaghan TM Pakpour S Severino A Kuijper EJ. Fecal microbiota transplantation: current challenges and future landscapes. Clin Microbiol Rev. (2024) 37:e0006022. 10.1128/cmr.00060-22 38717124 PMC11325845

[4] Taghizadeh Ghassab F Shamlou Mahmoudi F Taheri Tinjani R Emami Meibodi A Zali MR Yadegar A. Probiotics and the microbiota-gut-brain axis in neurodegeneration: beneficial effects and mechanistic insights. Life Sci. (2024) 350:122748. 10.1016/j.lfs.2024.122748 38843992

[5] Zhao L-Y Mei J-X Yu G Lei L Zhang W-H Liu K. Role of the gut microbiota in anticancer therapy: from molecular mechanisms to clinical applications. Signal Transduct Target Ther. (2023) 8:201. 10.1038/s41392-023-01406-7 37179402 PMC10183032

[6] Nabavi-Rad A Sadeghi A Asadzadeh Aghdaei H Yadegar A Smith SM Zali MR. The double-edged sword of probiotic supplementation on gut microbiota structure in *Helicobacter pylori* management. Gut Microbes. (2022) 14:2108655. 10.1080/19490976.2022.2108655 35951774 PMC9373750

[7] Jiang S Ma W Ma C Zhang Z Zhang W Zhang J. An emerging strategy: probiotics enhance the effectiveness of tumor immunotherapy via mediating the gut microbiome. Gut Microbes. (2024) 16:2341717. 10.1080/19490976.2024.2341717 38717360 PMC11085971

[8] Lee M Chang EB. Inflammatory bowel diseases (IBD) and the microbiome-searching the crime scene for clues. Gastroenterology. (2021) 160:524–37. 10.1053/j.gastro.2020.09.056 33253681 PMC8098834

[9] Lamb CA Kennedy NA Raine T Hendy PA Smith PJ Limdi JK. British Society of Gastroenterology consensus guidelines on the management of inflammatory bowel disease in adults. Gut. (2019) 68:s1. 10.1136/gutjnl-2019-318484 31562236 PMC6872448

[10] Ma T Shen X Shi X Sakandar HA Quan K Li Y. Targeting gut microbiota and metabolism as the major probiotic mechanism - an evidence-based review. Trends Food Sci Technol. (2023) 138:178–98. 10.1016/j.tifs.2023.06.013

[11] Lin Y Wang J Bu F Zhang R Wang J Wang Y. Bacterial extracellular vesicles in the initiation, progression and treatment of atherosclerosis. Gut Microbes. (2025) 17:2452229. 10.1080/19490976.2025.2452229 39840620 PMC12934168

[12] Yadegar A Pakpoor S Ibrahim FF Nabavi-Rad A Cook L Walter J. Beneficial effects of fecal microbiota transplantation in recurrent *Clostridioides difficile* infection. Cell Host Microbe. (2023) 31:695–711. 10.1016/j.chom.2023.03.019 37167952 PMC10966711

[13] Kashyap PC Chia N Nelson H Segal E Elinav E. Microbiome at the frontier of personalized medicine. Mayo Clin Proc. (2017) 92:1855–64. 10.1016/j.mayocp.2017.10.004 29202942 PMC5730337

[14] Gulliver EL Young RB Chonwerawong M D'Adamo GL Thomason T Widdop JT. Review article: the future of microbiome-based therapeutics. Aliment Pharmacol Ther. (2022) 56:192–208. 10.1111/apt.17049 35611465 PMC9322325

